# Führen und Entscheiden unter Unsicherheit

**DOI:** 10.1007/s11612-021-00607-4

**Published:** 2021-11-02

**Authors:** Falko von Ameln

**Affiliations:** 1 Leipziger Str. 9, 26506 Norden, Deutschland; 2 Fakultät für Interdisziplinäre Forschung und Fortbildung (IFF), Alpen-Adria Universität Klagenfurt, Universitätsstr. 65–67, 9020 Klagenfurt, Österreich

**Keywords:** Corona, Covid-19, Entscheidung, Führung, HRO, Kontingenz, Kommunikation, Komplexität, Organisation, Organisationstheorie, Systemtheorie, Systemvertrauen, Unsicherheit, Vertrauen, Communication, Complexity, Contingency, Corona, Covid-19, Decision-making, HRO, Institution-based trust, Leadership, Organization, Organization theory, Systems theory, Trust, Uncertainty

## Abstract

Dieser Artikel der Zeitschrift „Gruppe. Interaktion. Organisation. (GIO)“ gibt einen Überblick über die Herausforderungen des Führens und Entscheiden unter Unsicherheit. Diese Herausforderungen werden am Beispiel des deutschen Corona-Managements illustriert und auf Problemlagen in organisationalen Entscheidungsprozessen bezogen. Der Text verfolgt vorrangig den Anspruch, Dilemmata des Entscheidens in einer Kombination organisationspsychologischer und organisationssoziologischer Perspektiven herauszuarbeiten und vor dem Hintergrund sozialer Kontextbedingungen aufzuschließen, gibt aber auch Empfehlungen für die Gestaltung einer Organisations- und Führungskultur, die den Anforderungen des Entscheidens unter Unsicherheit Rechnung trägt.

## Abstract (en)

This article in the journal “Gruppe. Interaktion. Organisation. (GIO)” summarizes the challenges of leadership and decision making under uncertainty. These challenges are illustrated using the example of corona management in Germany and related to problems in organizational decision-making processes. The main aim of the text is to shed light on the dilemmas of decision-making using a combination of organizational psychological and organizational sociological perspectives and to explain these dilemmas with reference to the social context of decision making. The final section gives recommendations for an organizational and leadership culture that meets the requirements of decision-making under uncertainty.

Wie anspruchsvoll Führen und Entscheiden unter Unsicherheit sind, hat uns der Blick auf die politischen Entscheidungsprozesse im Zuge des Corona-Managements deutlich vor Augen geführt. Hier zeigte sich, unter welchem Druck Entscheider*innen in einer von Ungewissheit und konfligierenden Erwartungen geprägten Situation stehen. Es zeigte sich aber auch, wie Zielkonflikte und Paradoxien, unzureichend geklärte Entscheidungsverfahren und eine eher regelungsorientierte Kultur eine lösungsorientierte und konsensfähige Entscheidungsfindung erschweren.

In der viel zitierten VUKA-Welt (Mack et al. 2015), also von Volatilität, Unsicherheit, Komplexität und Ambiguität geprägten Organisationsumwelten, werden Krisendynamiken (d. h. von hohem Veränderungsdruck geprägte, existenzbedrohende Situationen ohne bekannten Lösungsweg) häufiger, wenngleich sie meist weniger dramatisch und öffentlichkeitswirksam in Erscheinung treten. Die Pandemie zeigt dabei in zugespitzter Form die Herausforderungen auf, vor denen Führung in *jeder* unsicherheitsbesetzten Entscheidungssituation steht, nicht nur in der Politik, sondern in Organisationen jeglichen Typs.

Ausgehend von dieser These, die im ersten Abschnitt belegt werden soll, versucht der vorliegende Artikel, fünf generelle Herausforderungen des Führens und Entscheidens unter Unsicherheit aufzuzeigen. Dabei soll die Komplexität der Beobachtungen auf der Phänomenebene zumindest in Teilen mithilfe psychologischer und soziologischer Erklärungsansätze aufgeschlossen und in ihren Zusammenhängen dargestellt werden^1^.

## Herausforderung 1: Entscheiden trotz Ungewissheit und Nichtwissen

Was ist eine „gute“ Entscheidung? Die Frage unterstellt, dass es angebbare normative Bedingungen des Entscheidens gäbe. Eine Entscheidung ist in dieser Perspektive der klassischen Entscheidungstheorie dann „gut“, wenn sie dazu beiträgt, zwischen verschiedenen Alternativen diejenige auszuwählen, die den vorab definierten Kriterien am ehesten gerecht wird, z. B. dem definierten Ziel am nächsten kommt, ein optimales Kosten-Nutzen-Verhältnis aufweist usw. Dass Entscheidungen in Organisationen keineswegs so lehrbuchmäßig rational verlaufen, ist nicht nur aus der Praxis bekannt, sondern auch von der Organisationsforschung vielfach beschrieben worden. So hat March sehr pointiert darauf hingewiesen, dass Entscheidungsgelegenheiten, Lösungen und Akteure in Organisationen nur lose gekoppelt sind und dass Entscheidungsprozesse daher nicht der klassisch-rationalistischen Ordnungsvorstellung entsprechen, sondern eher der Ordnung in einem Mülleimer (March und Olsen 1986) gleichen. Unsicherheit in Entscheidungsprozessen entsteht aber nicht erst in dieser Gemengelage „organisierter Anarchien“ (Cohen und March 1974), sondern ist von einer grundlegenderen Natur.

Abgesehen von Idealbedingungen sind Entscheidungen immer mit verschiedenen Formen von Unsicherheit^2^ konfrontiert. In manchen Entscheidungssituationen sind zwar die möglichen zukünftigen Zustände und auch ihre statistische Eintretenswahrscheinlichkeit bekannt. Beim Roulette liegt die Wahrscheinlichkeit für „rot“ bei 50 %, es ist aber unsicher, ob eine rote oder eine schwarze Zahl fallen wird. Diese Form der Unsicherheit wird aleatorische Unsicherheit (ebd., S. 119) oder *Risiko* genannt. Wenn zukünftige Zustände bekannt, deren Eintretenswahrscheinlichkeiten aber unbekannt sind, hat man es mit einer anderen Form von Unsicherheit zu tun, die Apelt und Senge (2015, S. 2) im Anschluss an Dequech als *Ungewissheit* bezeichnen.

Entscheider*innen in komplexen Entscheidungssituationen haben es nicht nur mit Risiko und Ungewissheit zu tun, sondern mit einer noch weiter reichenden Art von Unsicherheit, die aus den Eigenschaften komplexer Systeme (Dörner und Schaub 1995) resultieren – hier veranschaulicht am Beispiel der Corona-Pandemie:

- *Neuartigkeit* (Zu Beginn der Pandemie war vom „neuartigen Corona-Virus“ die Rede. Auch heute, über ein Jahr später, werden wir von der Entwicklung immer wieder überrascht.)
- *Offenheit der Zielsituation* (Das Ziel der Corona-Schutzmaßnahmen – „flatten the curve“/Vermeidung einer Überlastung der Intensivstationen/Null-Covid/Herdenimmunität … – steht nicht fest, sondern musste und muss immer wieder neu diskursiv entwickelt und konsentiert werden.)
- *Polytelie* (Hinzu kommt, dass unterschiedliche und z. T. widersprüchliche Ziele miteinander konkurrieren, z. B. Verringerung der Infektionszahlen vs. Öffnung der Schulen – siehe Abschnitt 2)
- *Vielzahl der Variablen* (Der Pandemieverlauf hängt von der Infektiosität der Virus-Mutationen, der Compliance der Bevölkerung, der Verfügbarkeit von Impfstoffen und Medikamenten, der Arbeit der Gesundheitsämter, der Außentemperatur und zahllosen weiteren Faktoren ab, die nicht alle bekannt und in ihrer Interdependenz erforscht sind.)

Ein zentrales Problem in komplexen Entscheidungssituationen ist also der Umgang mit Nichtwissen. Beispielsweise wurde und wird diskutiert, ob Geimpfte das Virus noch übertragen können. Während dieses *spezifische Nichtwissen* (also das bekannte Nichtwissen: Man weiß, dass man etwas nicht weiß) bei den Entscheider*innen das Erleben von Unsicherheit erzeugt, ist für das Management komplexer Entscheidungssituationen das *nicht-spezifische Nichtwissen* (also das, wovon man nicht weiß, dass man es nicht weiß) noch sehr viel problematischer. Ein weiteres Problem sind die zahlreichen typischen *Denkfehler* im Umgang mit komplexen Systemen (vgl. Schaub 2006), z. B. Vernachlässigung der Systemdynamik im Zeitverlauf, linear-kausales Denken unter Vernachlässigung der Wechselwirkungen, wishful thinking, Bekämpfung der Symptome anstatt der Ursachen, um nur einige wenige zu nennen. Hinzu kommt eine ebenso große Anzahl kognitionspsychologisch begründeter Urteilsfehler beim Entscheiden (base rate neglect, overconfidence u. v. a., vgl. Pfister et al. 2017, S. 149 ff.; Walz 2015).

Sowohl externe als auch interne Unsicherheiten führen also dazu, dass mögliche zukünftige Zustände des Systems nicht linear aus dessen gegenwärtigem Zustand ableitbar sind. Dies bringt eine qualitativ anders gelagerte Form der Unsicherheit mit sich, die Dequech (2011) als *fundamentale Unsicherheit* bezeichnet.

Im saturierten, kontinuitäts- und wachstumsgeprägten Umfeld der Moderne konnten sich in den Industrienationen viele Organisationen der angenehmen Illusion hingeben, dass ihre Umwelten Entscheidungsfindung, strategisches Management und Geschäftsentwicklung unter der Grundannahme von Stabilität und Rationalität ermöglichen. Spätestens die Corona-Pandemie hat uns vor Augen geführt, dass fundamentale Unsicherheit nicht nur in besonders dynamischen Branchen wie der IT, sondern in allen organisationalen Umfeldern auftritt und sich lediglich in Abhängigkeit von Organisationstyp, Branche, Geschäftsmodell usw. unterschiedlich ausprägen. Denn Volatilität, Unsicherheit, Komplexität und Ambiguität werden nicht nur vom Marktumfeld getrieben, sondern auch von Fachkräftemangel und Wertewandel (v. Ameln und Wimmer 2016) – oder eben von einer unerwarteten Pandemie.

Nicht Sicherheit – wie von der klassischen Entscheidungstheorie postuliert – sondern Unsicherheit ist also der Normalfall in Entscheidungssituationen: „Jede Organisation operiert in einer Welt, die sie nicht kennen kann. Diese Welt wird durch Unsicherheitsabsorption in eine bekannte Welt überführt, durch eine bekannte Welt ersetzt“ (Luhmann 2015, S. 35). So wird fundamentale Unsicherheit in Risiko transformiert: Zwar weiß man nicht, wie sich die Dinge entwickeln, kann aber handeln – in aller Vorläufigkeit und auf die Gefahr hin, dass die Entscheidung sich später als falsch erweist. Ob die Entscheidung dabei rational, zielführend usw. ist, liegt im Auge des Beobachters, ist aber keine Voraussetzung für das Entscheiden selbst.

## Herausforderung 2: Umgang mit Paradoxien und Multirationalitäten

In der Pandemie-Bekämpfung ist offenkundig geworden, dass in komplexen Entscheidungssituationen verschiedene inkommensurable Situationsdefinitionen, Werte und daraus resultierende Zielsetzungen aufeinandertreffen:

- Gesundheitsschutz vs. Freiheitsrechte
- bundesweit einheitliche vs. lokal angepasste Regeln
- Langfriststrategie vs. tagesaktuelle Reaktion
- Schnelligkeit vs. Gründlichkeit bei der Zulassung vom Impfstoffen
- Impfung besonders vulnerabler Gruppen vs. Impfung von Menschen mit vielen Kontakten
- nationale vs. universalistische Verantwortung bei der Beschaffung von Impfstoffen

Das Zusammentreffen dieser unterschiedlichen Logiken und Rationalitäten führt zu widersprüchlichen Anforderungen an Entscheidungen und damit zu Dilemmasituationen für die Entscheider*innen, wie sie Robert Habeck, Bundesvorsitzender der Grünen, im Herbst 2020 mit Blick auf die ansteigenden Fallzahlen benennt: „Im Frühjahr hatten wir nur ein Ziel: eine Triage in den Krankenhäusern zu verhindern. Jetzt haben wir mindestens zwei Ziele, wir wollen eine Triage und einen Lockdown verhindern. Aber wir wissen nicht, ob sich diese beiden Ziele ausschließen“ (Gloger und Mascolo 2021, S. 287).

Die Spannungsfelder im Corona-Management illustrieren in zugespitzter Form die Spannungsfelder, mit denen sich Entscheidungsfindung in Organisationen generell konfrontiert sieht. Die Vorstellung, dass Organisationen einem konsistenten Organisationsziel und einer sich daraus ableitenden einheitlichen Rationalität folgen könnten, ist von der Organisationstheorie längst ad acta gelegt worden. Stattdessen müssen Organisationen immer konkurrierende Logiken ausbalancieren, z. B.

- verschiedene gesellschaftliche Funktionslogiken (etwa stehen Gesundheitsämter bei der Bewältigung der Corona-Pandemie nicht nur im Spannungsfeld von Politik, Wissenschaft und Recht, sondern sind auch ökonomischen Zwängen unterworfen),
- Binnenlogik der Organisation vs. Kundenlogik (d. h. Erwartungen der Kund*innen),
- verschiedene Professionslogiken (z. B. unterschiedliche Behandlungsansätze, die sich für eine Reha-Patientin aus ärztlicher/psychologischer/ergotherapeutischer Sicht ergeben),
- Professionslogiken vs. Organisationslogik (das fachlich Gebotene ist nicht immer im Interesse der Gesamtorganisation),
- interne Funktionslogiken (z. B. Einkauf vs. Produktion),
- divisionale Logiken (Zentrale vs. Untereinheiten),
- regionale Logiken
- usw.

Solche Multirationalitäten sind in der Organisationstheorie vorrangig unter dem Begriff der *Paradoxie* thematisiert worden^3^. Wie aus dem bisher Gesagten deutlich wird, sind Paradoxien in Organisationen also kein Sonderfall, sondern ergeben sich unausweichlich aus dem Zusammentreffen lokaler Rationalitäten, die durch System-Umwelt-Differenzierungsprozesse auf verschiedenen Ebenen entstehen (vgl. Smith und Lewis 2011). Damit wird die Notwendigkeit eines Umgangs mit Paradoxien zu einer Grundkonstante des Entscheidens in Organisationen schlechthin (Luhmann 2015). Die Komplexitätssteigerung organisationaler Umwelten (Stichwort „VUKA“) bringt es mit sich, dass solche Paradoxien des Entscheidens sich potenzieren oder zumindest weniger leicht ignoriert werden können. Infolgedessen, so Luhmann (ebd., S. 45), „wird es nötig werden, die Entscheidungstheorie selbst von Prinzip auf Paradoxie umzustellen“.

Schnelles, eindeutiges Entscheiden, Zupacken, Probleme lösen – das entspricht dem Rollenbild der heroischen Führungskraft, dem Anspruch, den viele Führungskräfte an sich selbst haben und oftmals auch den Erwartungen aus dem System. Paradoxe Entscheidungssituationen werden in dieser Motivlage erst einmal als störend, hemmend, frustrierend empfunden: Wie man es macht, ist es falsch. In der Pandemie ist zu beobachten, dass totaler Lockdown eine ebensowenig gangbare Alternative ist wie totale Öffnung. Deshalb, so Simon (2007, S. 75), „führt keine Führungskraft ihren Job optimal aus, wenn sie dauerhaft Eindeutigkeit schafft“. „Komplexitätsvergessene Vernunft“ (Nassehi 2018), die eine Seite der Paradoxie und damit einen Teil der Komplexität systematisch ausblendet, geht den Denkfehlern im Umgang mit komplexen Systemen auf den Leim. Führungskonzepte, die dem paradoxen Charakter von Entscheidungssituationen Rechnung tragen, kommen u. a. unter dem Stichwort der ambixdextren Führung (z. B. Szlang und Bruch 2020) stärker in den Blick, allerdings verdient dieses Themenfeld von den Organisationswissenschaften noch stärkere Aufmerksamkeit.

## Herausforderung 3: Akzeptanz schaffen trotz widersprüchlicher Erwartungen

Die psychologische Tradition versteht Entscheidung „als einen mentalen Prozess, dessen zentrale Komponenten Beurteilungen (judgments), Bewertungen (evaluations) und Wahlen (choices) sind […]. Der Prozess endet, wenn sich eine Person durch die Wahl einer Option festlegt, oder mit der tatsächlichen Umsetzung der getroffenen Wahl“ (Pfister et al. 2017, S. 3 f.). Um den Fokus der klassischen Entscheidungstheorie auf den Entscheider und seinen „mentale Entscheidungsprozess“ zu weiten, muss man die sozialen Prämissen des Entscheidens, ihre Folgewirkungen im sozialen System und die Rückwirkungen, die diese antizipierten Folgewirkungen auf den Entscheider haben, stärker mitberücksichtigen. Vor dieser Anforderung stehen nicht nur die Forschung, sondern auch die Entscheider*innen selbst. So kann eine Entscheidung aus der Sicht des Entscheiders rational richtig, aber dennoch aus Führungsperspektive „falsch“ (i. S. v. „nicht umsetzbar“, „kontraproduktiv“) sein, wenn die Entscheidung keine soziale Akzeptanz erfährt und nicht gemeinsam umgesetzt wird.

Entscheiden ist in diesem Verständnis ein komplexer sozialer Koordinierungsprozess, der weitaus mehr beinhaltet als die Wahl zwischen alternativen Handlungsmöglichkeiten. Brunsson (1990, S. 48) formuliert es so:

Organizations have more problems than choice. Another fundamental organizational problem is to achieve co-ordinated, collective action. It has been argued that decision-making and decisions are sometimes used for solving that problem, and that this affects the design of the decision process […]. In order to mobilize organizational action, it is important to secure commitment from presumed actors […]. Decision-making may be used as a way of forming commitments, both directly and indirectly.

Eine „gute“ Entscheidung ist in diesem Sinne also nicht unbedingt eine, die die gemessen an „rationalen“ Kriterien beste Alternative auswählt, sondern eine, die soziale Akzeptanz und Folgebereitschaft herstellt. Diese Anforderung an Entscheidungen, die gerade Führungskräften vertraut ist, weist auf die Bedeutung der Einbindung der Betroffenen in Entscheidungsprozesse, der Begründung von Entscheidungen, der kommunikativen Einkleidung von Entscheidungen und des Impression Managements auf der Seite der Entscheider*innen hin.

Aus systemtheoretischer Sicht ist jede Entscheidung mit dem Problem der doppelten Kontingenz konfrontiert: Diejenigen, die die Entscheidung umsetzen sollen, können im Sinne der getroffenen Entscheidung handeln (z. B. die Maskenpflicht beachten), aber auch etwas anderes oder das Gegenteil tun. Schlimmstenfalls tun sie das Gegenteil, gerade *weil* so entschieden wurde. Solche Reaktanzreaktionen dürften gerade bei selbsternannten „Querdenkern“ (also den „Widerstandskämpfern“ in den gängigen Modellen zu Haltungen in Change-Prozessen), ab einem gewissen Grad der Corona-Müdigkeit aber auch bei zuvor Gutwilligen die Akzeptanz beeinflussen. An dieser Stelle zeigt der Blick auf die Pandemie wiederum ein Dilemma, mit denen auch Entscheider*innen in Organisationen konfrontiert sind: Manches (z. B. Schließung des Einzelhandels) lässt sich erzwingen, Vieles aber nicht, und gerade diese nicht erzwingbaren, sondern nur freiwillig zu erbringenden Leistungen („nicht intendierbare Leistungen“, Elster 1987, S. 141 ff.) wie z. B. die Einhaltung von Hygieneregeln im privaten Raum sind oft entscheidend für die Erreichung der mit der Entscheidung angestrebten Ziele. Da die Abhängigkeit von solchen nicht intendierbaren Leistungen die Macht der Führung begrenzt (v. Ameln und Kramer 2012; v. Ameln und Heintel 2016, S. 121 ff.), müssen Entscheidungen und die Kommunikation von Entscheidungen immer auch auf das Akzeptanzmanagement hin ausgerichtet werden. Die daraus abgeleiteten Entscheidungsprämissen stehen oft quer zu fachlichen Kriterien der Rationalität, was im Corona-Management sicherlich einer der Gründe für das viel kritisierte „Herumeiern“ der Politik und die Vielstimmigkeit der Ministerpräsident*innen war.

Ein in Entscheidungssituationen zentraler Mechanismus der Unsicherheitsreduktion ist *Vertrauen* (Luhmann 2014). Vertrauen kann unterschiedliche Bezugspunkte haben. Man kann Vertrauen in die *persönliche Integrität* der Person haben, die eine Entscheidung trifft – dann vertraut man darauf, dass diese Person die Entscheidung zum Wohle der Allgemeinheit, im Sinne der Systemrationalität usw. und damit weitgehend frei von persönlichen Einzelinteressen trifft. Vertrauen kann sich auf die *Kompetenz* des Entscheiders oder der Entscheiderin beziehen. Karl Lauterbach wurde in der Pandemie vor allem deswegen so viel Vertrauen entgegengebracht, weil ihm als Mediziner die für eine wirksame Corona-Politik notwendige Fachkompetenz zugeschrieben wurde. Man kann *Vertrauen in ein Entscheidergremium* haben, also darin, dass die Beteiligten durch gute Kooperation und Abstimmung zu guten Entscheidungen kommen. Auch diese Form von Vertrauen erscheint im deutschen Pandemie-Management zunehmend gestört: Vor allem durch Alleingänge der Ministerpräsident*innen, die gemeinsam getroffene Vereinbarungen immer wieder hinterliefen, „verfestigt sich der Eindruck politischer Vielstimmigkeit, des Sowohl-als-auch, das viele als „die da oben wissen nicht, was sie wollen“ wahrnehmen“ (Gloger und Mascolo 2021, S. 253). Der Eindruck, dass die Verantwortlichen eher gegeneinander als miteinander arbeiten, kulminierte im Konflikt um die CDU-Kanzlerkandidatur. Dass die Grünen in den Meinungsumfragen wenig später die CDU überholten, darf als Indiz dafür gesehen werden, welchen Wert Menschen darauf legen, dass sich die Verantwortungsträger als einig und abgestimmt präsentieren.

Solches personales Vertrauen in Personen und Entscheidergremien ist in Organisationen und Gesellschaft wichtig, aber auf Dauer nicht entscheidend, da die Personen in Politik und Organisationen wechseln. Problematischer ist, dass die Corona-Krise eine Krise des *Systemvertrauens* offenbart, d. h. des Vertrauens in das Funktionieren von Institutionen, die Einhaltung von Regeln, die Anwendung definierter Standards und die durchgängige Beachtung von Kontrollmechanismen. Gefühle des „Abgehängtseins“, des Verlusts von Diskursmacht und der erlebten Delegitimierung der eigenen Sprecherposition begünstigen offenbar die Erosion von Systemvertrauen, wie nicht erst bei der sogenannten „Querdenker“-Bewegung, sondern bereits im Fall der politischen Polarisierung in den USA offenkundig wurde^4^. Daraus lässt sich die These ableiten, dass Systemvertrauen in Situationen von Unsicherheit und Umbruch eine besonders wichtige Rolle spielt. Im Sinne dieser These fanden Bäuerle et al. (2020) in der bereits in Abschnitt 2 erwähnten Studie signifikante negative Korrelationen zwischen dem Vertrauen in das Corona-Management der deutschen Bundesregierung und generalisierten Angstsymptomen (r = −0,153, *P* < 0,001) sowie psychologischem Belastungserleben (r = −0,154, *P* < 0,001). Von einem solchen Zusammenhang zwischen Systemvertrauen, Unsicherheit und Angst dürfte man nicht nur in Bezug auf gesellschaftliche Transformationen (z. B. den gesellschaftlichen Umbruch von der Industrie- zur Wissensgesellschaft), sondern auch auf Transformationsprozesse in Organisationen ausgehen können. Die Betonung der Relevanz von Systemvertrauen wird durch die Forschung zur Bedeutung prozeduraler Gerechtigkeit für Führung und Change-Prozesse (z. B. Streicher und Frey 2012) gestützt.

Für Führen und Entscheiden unter Unsicherheit sowie für die Gestaltung von Veränderungsprozessen leitet sich aus diesen Überlegungen die Erkenntnis ab, dass die Akzeptanz von Entscheidungen vom Vertrauen in die Akteure abhängt. Diesbezügliche Empfehlungen wie eine möglichst intensive persönliche Kommunikation und ein Bemühen um interpersonelle Fairness gehören zum Standardrepertoire der Führungslehre. Vielleicht noch ausschlaggebender, aber in Organisationen oft weniger beachtet ist das Systemvertrauen. In dieser Hinsicht sind verbindliche Verfahren, die Konsistenz der Einhaltung dieser Verfahren und die Einbindung der Betroffenen in Entscheidungsprozesse von Bedeutung (wobei hier weniger ein Mitentscheidungsrecht als die Möglichkeit, sich mit persönlichen Meinungen und Kritik zu äußern = „voice“ als bedeutsam benannt wird, vgl. z. B. Oswald 2010). Von der Demokratietheorie können wir zusätzlich lernen, dass Mechanismen der Selbstrelativierung von Macht („checks & balances“) wichtig sind, um Systemvertrauen zu schaffen und zu erhalten.

## Herausforderung 4: Entscheidungsverfahren professionalisieren

Das gemeinsame Treffen und Umsetzen von Entscheidungen wird in der Öffentlichkeit derzeit vielleicht als größte Schwachstelle der Corona-Politik wahrgenommen. Dabei sind zwei Ebenen zu unterscheiden:

- die einzelne Entscheidung selbst und
- das Verfahren (oder, mit Luhmann: das Programm), mit dem Entscheidungen getroffen werden.

Wie Entscheidungen angefertigt werden, ist in wichtigen Fällen über ein *Programm* definiert: Im Gesetzgebungsverfahren ist beispielsweise festgelegt, welche Verfassungsorgane mit welchen Rollen und Rechten in welcher Reihenfolge an der Entstehung und Verabschiedung von Gesetzen beteiligt werden. Im der dritten Welle der Pandemie wurde das Fehlen eines Programmes beklagt, das Kompetenzstreitigkeiten zwischen Bund und Ländern effektiv regeln würde (was schließlich zur Aufnahme der „Bundesnotbremse“ in das Infektionsschutzgesetz am 21.04.2021 führte). Die dadurch entstehenden Verwerfungen (also Kompetenzgerangel, manifeste Konflikte, heftiger Streit über Entscheidungskriterien) zeigten sich in ähnlicher Form bei der Entscheidung über die Kanzlerkandidatur in der CDU. Hier manifestiert sich ein Defizit, das auch in vielen Organisationen zu beobachten ist: Zwar wird permanent entschieden, aber die Art der Entscheidungsfindung ist aufwändig, konfliktträchtig, unsystematisch und oft nicht zielführend (March und Olsen 1986; siehe auch Abschnitt 1). Gerade angesichts des Umstands, dass Entscheiden das Kerngeschäft der Organisation darstellt, ist bemerkenswert, dass Entscheidungsprogramme oft eher als informelle Gepflogenheiten historisch aus der Praxis erwachsen als dass sie formal entschieden würden. Zwar sind Verantwortlichkeiten (z. B. in Geschäftsverteilungsplänen) festgeschrieben, mit der jeweiligen Stelle verbundene Entscheidungsbefugnisse definiert und Entscheidungsgremien etabliert. Man weiß beispielsweise, dass strategische Entscheidungen im Rahmen der Vorstandssitzung zustande kommen, aber nach welchen Entscheidungsprinzipien innerhalb des Vorstandsgremiums entschieden wird, inwieweit andere Verantwortliche innerhalb der Organisation einbezogen werden usw., ist oft nicht klar geregelt. Dieses Regelungsdefizit kann insofern funktional sein als eine flexible Anpassung des Entscheidungsmodus an die Gegebenheiten ermöglicht: Je nach Entscheidungsanlass kann man mehr oder weniger Zeit und Sorgfalt auf die Entscheidung verwenden und mehr oder weniger Akteure einbeziehen; man kann miteinander diskutieren und wenn sich kein Konsens einstellt, kann die Führungskraft das Thema immer noch zur „Chefsache“ erklären.

Häufig produziert diese Flexibilität aber Nebenfolgen, wie sie auch beim Corona-Management zu beobachten sind: Entscheidungsprozesse werden träge und laden sich mikropolitisch auf, Entscheidungen werden in Frage gestellt, wenn sich Betroffene exkludiert fühlen (vgl. die obenstehenden Hinweise zu prozeduraler Gerechtigkeit).

Diese Probleme lassen sich nicht gänzlich ausräumen, aber doch reduzieren. Organisationen müssen entscheiden, wie entschieden wird. Im Zuge der Agilitätsdebatte wurden Entscheidungsverfahren entwickelt (die in basisdemokratischen Initiativen im Übrigen schon lange in ähnlicher Form praktiziert wurden), die die inhaltliche Qualität und die Akzeptanz von Entscheidungen steigern sollen. Eine Entscheidungsarchitektur kann darüber Auskunft geben, welcher Entscheidungsanlass welche Anforderungen an die Entscheidungsfindung mit sich bringt und welches Verfahren diese Anforderungen am besten erfüllt, darunter z. B. kollegiale Rollenwahl, um die Person zu ermitteln, die die höchste fachliche Kompetenz für die Entscheidung aufweist, die Konsent-Moderation, um Einwände qualitativ zu minimieren und die Widerstandsabfrage, um Einwände quantitativ zu minimieren.

Das bedeutet keineswegs, dass Entscheidungen in komplexen Dilemmasituationen immer im Einklang getroffen werden könnten. Eigene Betroffenheiten erschweren eine Entscheidung im Sinne des Allgemeinwohls (der Systemrationalität). An diesen Stellen hat Führung das Recht und die Funktion, Entscheidungen auch ohne Zustimmung der übrigen Systemmitglieder zu treffen (Luhmann 1976, S. 215). Oftmals werden solche „harten“ Entscheidungen auch von den Betroffenen erwartet. Gleichzeitig endet das hierarchische Durchgriffsrecht der Führung aber immer an den Grenzen der Folgebereitschaft der Geführten und an der Stelle, an der Vertrauen und Systemvertrauen zu erodieren beginnen (siehe der vorangegangene Abschnitt). Führung als (auf der informellen, nicht der formalen Ebene) vom System delegierte Autorität steht also immer in einem gegenseitigen Bedingungsverhältnis von prozeduraler Gerechtigkeit und hierarchischen Sonderbefugnissen. Die gemeinsam getragene Festlegung von Entscheidungsverfahren ist also eine Vorbedingung dafür, dass Führung in diesem komplexen Wechselspiel von symmetrischer und asymmetrischer Entscheidungsfindung Alleinentscheidungsrechte überhaupt wirkungsvoll wahrnehmen kann.

## Herausforderung 5: Eine Kultur für den kollektiven Umgang mit Unsicherheit etablieren

„Die Pandemie hält unserem Land den Spiegel vor. Der Hang zum Alles-Regeln-Wollen, unsere Angst vorm Risiko, das Hin- und Herschieben von Verantwortung – wie wir das ändern und wie wir auch unsere Institutionen krisentauglicher machen, all das wird aufzuarbeiten sein.“ Ähnliche dysfunktionale kulturelle Muster, wie sie Frank-Walter Steinmeier in seiner Osteransprache vom 03.04.2021 kritisiert, behindern nicht nur eine effiziente Pandemiebekämpfung auf politischer Ebene, sondern sind auch in vielen Organisationen zu finden. Auch Gerd Gigerenzer, bekannt durch seine Forschung zu Risiko und Entscheidungen, konstatiert in der Pandemie eine Scheu vor der Übernahme von Verantwortung und meint: „Wir bewegen uns immer mehr von einer Leistungsgesellschaft auf eine Absicherungsgesellschaft zu“ (Gigerenzer und Kittlitz 2021, S. 61). Dieser Abschnitt beschäftigt sich daher mit der Frage welche kollektiven „mindsets“ und Kulturmerkmale für das Entscheiden in unsicherheitsbehafteten Situationen hilfreich sein könnten. Die Forschung zu Organisationen, die in besonderem Maße darauf angewiesen sind, unter Bedingungen von Volatilität, Unsicherheit und Komplexität hoch zuverlässige Entscheidungen zu treffen (High Reliability Organizations/HRO, vgl. Weick und Sutcliffe 2017; also z. B. Atomkraftwerke, Flugzeugträger oder chirurgische Abteilungen in Krankenhäusern), bietet hier interessante Perspektiven.

Gebauer (2017, S. 243 ff.) entwickelt – aufbauend auf Vorarbeiten von Westrum und Hudson – ein 5‑Stufen-Modell für den Umgang mit Risiken. Das Modell soll hier in Kurzform dargestellt, mit weiterführenden Überlegungen angereichert und am Beispiel des Corona-Managements illustriert werden, um daraus Erkenntnisse für Führen und Entscheiden unter Unsicherheit abzuleiten.

### Stufe 1: Gleichgültiges Muster.

Auf dieser Stufe werden mögliche unerwartete Ereignisse weitgehend ausgeblendet. Dieses Muster herrschte offenbar vor der Pandemie in der Politik vor. Ressourcen zur Pandemiebekämpfung (also Vorräte an Schutzausrüstung, Personal, aber auch institutionelle Resilienz auf der Ebene von Know How, Kommunikation und Entscheidungen) wurden nicht aufgebaut oder sogar abgebaut, obwohl das Risiko einer Pandemie durchaus bekannt war. Eine Pandemie, so der Finanzmathematiker Nassim Nicholas Taleb im Interview mit Gloger und Mascolo (2021), sei kein „schwarzer Schwan“, also ein Ereignis mit extrem niedriger Eintretenswahrscheinlichkeit, sondern „das Gegenteil eines „schwarzen“ Schwans […], ein „weißer Schwan“ vielmehr – ein Ereignis nämlich, das mit Sicherheit eintreten wird“ (S. 86). Gloger & Mascolo beschreiben ausführlich, dass das Risiko einer Pandemie der Politik durchaus bewusst war, dass notwendige Maßnahmen als Konsequenz dieser Erkenntnis aber ausblieben. So hatte Angela Merkel schon 2015, nach der Ebola-Epidemie, grundlegende Reformen zur Verbesserung des Pandemiemanagements der WHO eingefordert. 2017 wurde im Vorfeld des G20-Gipfels unter deutschem Vorsitz mit den Gesundheitsministern ein zweitägiges Planspiel durchgeführt – das Szenario: Ein Virus, das zu Husten und Ersticken führt, bricht in einem Land namens „Anycountry“ (gemeint war China) aus und gerät außer Kontrolle (ebd., S. 81 f.). Ähnliche Erkenntnisse zeigten sich schon 2005, als 7 Bundesländer, 11 Ministerien und 50 Unternehmen den Umgang mit einer Influenzapandemie übten:

Das Szenario stammte vom RKI. Das Planspiel habe wichtige Erkenntnisse vor allem hinsichtlich der „psychologischen Reaktion der Bevölkerung in Krisensituationen“ gebracht, hieß es im Abschlussbericht. Entscheidungsträger seien auf die psychosozialen Folgen einer Pandemie nicht ausreichend vorbereitet […]. Der Bericht wies auf strategische Defizite etwa bei der Versorgung mit Schutzausrüstung hin. Und dabei blieb es.

Und die Frage nach den Zuständigkeiten im Katastrophenfall blieb – und bleibt – unbeantwortet: Wer darf was in einer Krise? (ebd., S. 95)

Wie die Beispiele zeigen, dürfte das, was Gebauer als „Gleichgültigkeit“ bezeichnet, auf typischen Denkfehlern im Umgang mit komplexen Systemen wie wishful thinking, Planoptimismus („wird schon gut gehen“) und Ablenkbarkeit durch andere Themen (Schaub 2006; s. oben) beruhen – psychoanalytisch könnte man von Verdrängung und Verleugnung sprechen.

Vor dem Hintergrund der in Abschnitt 1 eingeführten Unterscheidungen ist bemerkenswert, dass das Kernproblem beim Umgang mit Unsicherheit im Fall von Covid-19 also nicht das Nichtwissen war, sondern der Umgang mit einem vorhandenen Wissen, das man aber als schwaches Signal ignorieren konnte.

In einer Welt, die sich lange als stabil auffassen ließ, musste man zumindest scheinbar keine Strategien für den Umgang mit fundamentaler Unsicherheit entwickeln (einzelne Unternehmen und Branchen ausgenommen). Man konnte sich in Vorträgen von der rauen VUKA-Welt berichten lassen, dabei aber den eigenen blinden Fleck im Hinblick auf fundamentale Unsicherheit als Thema, das auch eine praktische Relevanz haben könnte, bewahren.

Man hängt gewissermaßen an den Resultaten langjähriger Unsicherheitsabsorption, die sich als bekannte Welt selbst validieren. Das gilt besonders, wenn man sich auf Risiken eingelassen hatte und es gut gegangen ist […]. In diesem Sinne gibt es zahllose Organisationen, die von ihren Mißerfolgen leben, weil gerade sie sichere Entscheidungsgrundlagen bieten. (Luhmann 2015, S. 36)

### Stufe 2: Reaktives Muster.

Auf Stufe 2 werden kompensatorische Maßnahmen ergriffen, wenn ein unerwartetes Ereignis eingetreten ist. Diese Maßnahmen bleiben aber ausschnitthaft und begrenzt. Wenn sich in einem Unternehmen beispielsweise ein Arbeitsunfall ereignet, wird man auf Stufe 2 eine Ursachenanalyse vornehmen, um diese Unfallursache zukünftig abzustellen (ohne allerdings andere Unfallrisiken systematisch in den Blick zu nehmen).

### Stufe 3: Berechnendes Muster.

Diese Stufe zeichnet sich durch den Versuch aus, Risiken durch Festlegung von Standards, Regeln und Prozessen in den Griff zu bekommen. Die Grundannahme ist, dass man Unsicherheit durch eine ausgefeilte lineare Steuerungslogik in den Griff bekommen kann: Das Ziel ist das Aufrechterhalten eines „Normalzustands“, zu jeder Abweichung lässt sich eine eindeutige Ursache feststellen, Abweichungen vom Plan werden mit der Nachbesserung des Plans beantwortet, der Mensch wird als Fehlerquelle betrachtet (Gebauer 2017, S. 91 ff.). Die Gefahr dieses Musters besteht darin, dass ab einer gewissen Regeldichte typische dysfunktionale Nebeneffekte formaler Organisationen entstehen: Angesichts eines unüberschaubaren Regelwerks tritt Verunsicherung auf, Regelbefolgung auf der Schauseite und informelles Verhalten im Verborgenen klaffen auseinander, Fehler werden vertuscht, die Verantwortung wird hin- und hergeschoben, Regeln werden nicht mehr ernstgenommen, die Achtsamkeit gegenüber Abweichungen nimmt ab – „Es entsteht die Illusion von Kontrolle bei gleichzeitigem Ansteigen der Ungewissheit“ (ebd., S. 82). Verbleibt man in dieser Logik, können sich also leicht dysfunktionale Muster einschleifen, die die organisationale Fähigkeit für den Umgang mit Unsicherheit dauerhaft einschränken, wie sie in der organisationstheoretischen Literatur (z. B. Ortmann 2004) vielfach beschrieben wurden.

Auf den Stufen 1 bis 3 werden Systeme als triviale Maschinen im Sinne von Heinz von Foerster konzipiert. Dieses Denken stößt durch die selbstverstärkenden Muster in Stufe 3 an eine Glasdecke (Gebauer 2017, S. 244 ff.), die nur durch eine andere Logik und ein komplexitätsadäquateres Verständnis von nicht-trivialen Systemen überwunden werden kann.

### Stufe 4: Proaktives Muster.

In Stufe 4 wird die lineare Logik der Stufen 1 bis 3 durch ein Verständnis ersetzt, das der Komplexität und Nicht-Linearität sozialer Systeme Rechnung trägt. Wesentlich sind dabei kulturell verankerte Praktiken der Selbstbeobachtung und -reflexion. Das Muster der Regelbefolgung wird (auch wenn Regeln natürlich weiterhin eine Rolle spielen) ergänzt durch ein Muster der kollektiven Achtsamkeit im Umgang mit Unsicherheit und ihren Auswirkungen auf das System. Gebauer beschreibt eine Vielzahl in HRO zu findender Praktiken, die vier Prinzipien dienen:

- unerwartete Ereignisse als Fenster zum System nutzen (z. B. mithilfe von Musteranalysen und Fragetechniken)
- Antizipieren (z. B. mithilfe von Ereignissimulationen oder der Entwicklung von Szenarien)
- Entwicklung von Resilienz (z. B. mithilfe agiler Planung oder strukturierter Verfahren zur Entscheidungsfindung)
- kontinuierliches Prüfen der Systemfitness (z. B. mithilfe von Kulturdialogen und Echtzeit-Stimmungsbildern)

Diese Praxis der kollektiven Achtsamkeit setzt ein hohes Maß an Kommunikation und Reflexion und damit organisational slack (d. h. Reservekapazitäten) voraus, der im Gesundheitssystem systematisch eliminiert wurde.

Entscheidungsprozesse sind in HRO nach Prinzipien organisiert, die agilen Organisationsprinzipien ähneln: Gerade in unsicherheitsbesetzten Situationen werden Entscheidungen von den Personen getroffen, die die meiste Erfahrung und das größte Wissen für die betreffende Situation aufweisen, ungeachtet der Frage, ob diese Personen auch formal zuständig sind (Senge und Dombrowski 2015). Entscheidungen in HRO sind oft netzwerkförmig organisiert. Intuition und implizites Wissen haben in Entscheidungsprozessen einen hohen Stellenwert – so sind Mitarbeitende in HRO dazu berechtigt, Prozesse auch auf der Basis eines bloßen Gefühls, dass etwas nicht stimmt, zu stoppen.

Kollektive Achtsamkeit im beschriebenen Sinne setzt drei wichtige Kulturmerkmale voraus, die systemische und agile Ansätze auch außerhalb von HRO zu kultivieren versuchen:

1. eine Nutzung interner Variabilität und Perspektivendifferenzen als Ressource für einen multiperspektivischen Zugang zu komplexen und mehrdeutigen Situationen. Aus der psychologischen Gruppenforschung ist seit langem bekannt, dass zu große Homogenität im Team eine komplexitätsadäquate Auseinandersetzung mit fundamental unsicheren Situationen erschwert (Groupthink). Für den Umgang mit VUCA-Situationen ist es daher empfehlenswert, Verschiedenartigkeit und Abweichungen im Team systematisch zu fördern.
2. die Fokussierung auf soziales Lernen und Selbstveränderung. Während auf den Stufen 1–3 die Annahme vorherrscht, dass unerwartete Ereignisse mit technischen Lösungen (z. B. verbesserter Kontroll- und Sicherheitstechnologie, Verfahrensoptimierung, Verfeinerungen des Regelsystems) in den Griff zu bekommen sind, setzen HRO auf Stufe 4 auf sozial-diskursive Prozesse der gemeinsamen Konstruktion von Wirklichkeit.
3. eine Kultur, in der Fehler und Abweichungen nicht als zu vermeidendes Problem, sondern als Lernchance verstanden werden. Als zentrales Element einer solchen Fehler- und Feedbackkultur wird psychologische Sicherheit (Edmondson 2020) verstanden. Edmondson unterscheidet drei Arten des Scheiterns: vermeidbares Scheitern (Scheitern wird – analog Stufe 3 – mit Sanktionen, Prozessoptimierung, Trainings usw. beantwortet), komplexes Scheitern (Verbesserung der Systeme auf der Basis einer Fehleranalyse aus mehreren Perspektiven) und intelligentes Scheitern. Bei Letzterem findet wiederum ein Umdenken statt: Ausgehend von der Erkenntnis, dass beim Umgang mit fundamentaler Unsicherheit Scheitern unvermeidlich ist und eine fehlervermeidende Kultur eher das kreative Denken und das Experimentieren mit unkonventionellen Lösungsansätzen behindert, wird Scheitern belohnt („failure awards“) und gefeiert („failure parties“).

Diese Tugenden waren in der deutschen Corona-Politik nur in Ansätzen zu beobachten.

Das bisherige Management der Pandemie lässt sich im Wesentlichen der Stufe 3 zuordnen (vgl. auch das einleitende Zitat des Bundespräsidenten). Die für Stufe 3 charakteristische Flut von Regeln und den Versuch, das Scheitern der regelbasierten Risikoeindämmung durch weitere, noch elaboriertere Regeln zu kompensieren, hat den öffentlichen Raum stark dominiert – die vorläufige mediale Kulmination war der oben schon erwähnte „Stufenplan“, aber auch Gesundheitsämter, Schulen und Unternehmen hatten große Mühe, die fast täglich wechselnden und erweiterten Verordnungen und RKI-Empfehlungen überhaupt zur Kenntnis zu nehmen. Wie auch in Organisationen häufig zu beobachten, hat sich die Sinnhaftigkeit vieler Regeln für viele nicht erschlossen, was zu einer Selbstentwertung des Regelsystems geführt hat.

Ein interdisziplinär besetzter „Corona-Rat“, der die Pluralität der Entscheidungslogiken in stärkerem Maße in politische Entscheidungsprozesse hätte einspiegeln können, ist zwar immer wieder angeregt, aber niemals realisiert worden. Überhaupt wurde die Pluralität der Perspektiven (so zumindest erschien es in den Medien) eher gegeneinander ausgespielt und nicht im Sinne der HRO-Prinzipien zunächst bewertungsfrei gewürdigt und als Ressource genutzt. So führten divergierende Positionen eher zu einem mit moralischem Unterton geführten Kampf um die Deutungsmacht, einer gegenseitigen Abwertung und einer zunehmenden Polarisierung der Debatte.

Dass in Deutschland weniger und später zu Verbreitungsmechanismen des Virus geforscht wurde als in anderen Ländern, wurde in der Öffentlichkeit kritisiert – dass die Antezendentien der Infektion wesentlich weniger im Fokus der Forschung zu stehen schienen als ihre Folgen, hat sicherlich z. T. wissenschaftsethische Gründe, lässt sich aber auch als Perpetuierung des Stufe 2-Musters „Troubleshooting ist wichtiger als umfassende Situationsanalyse“ lesen. Erfolgreiche Strategien anderer Länder kamen – zumindest in der öffentlichen Diskussion – kaum in den Blick (z. B. das nach der anfänglichen Verdrängungsstrategie ja offenbar recht erfolgreiche Corona-Containment in China) oder scheiterten an Regularien (wie eine wirksame Corona-App, die eines der zentralen Instrumente der erfolgreichen Corona-Politik in Taiwan war). Auch Modellversuche im eigenen Land (z. B. in Tübingen oder Rostock) wurden spät gestartet, im politischen Diskurs oft genug eher als moralisch prekäres Problem denn als Beitrag zur Lösung konnotiert und kaum im Hinblick auf nutzbare Erkenntnisse ausgewertet.

Ähnliche Kritik an der Corona-Politik anderer Staaten vor dem Hintergrund der HRO-Forschung äußern Mirvis (2020), Sanders (2020) und Schulman (2021).

### Stufe 5: Wertschöpfendes Muster.

Stufe 5 integriert die Praktiken und Kulturmerkmale der vierten Stufe, geht aber insofern über die vorangegangene Stufe hinaus als der kompetente Umgang mit Unsicherheit auf Stufe 5 als wesentlicher Beitrag zur Wertschöpfung der Organisation verstanden wird.

Wie eine Kultur der kollektiven Achtsamkeit im Umgang mit Unsicherheit in politischen Entscheidungsprozessen gefördert werden könnte, ist nicht Gegenstand dieses Artikels. Die Pandemie hat aber gezeigt, was passiert, wenn Systeme Umweltkomplexität mit Problemlösungsmustern zu bewältigen versuchen, die die soziale Binnenkomplexität des Systems nicht hinreichend berücksichtigen. Für Führen und Entscheiden unter Unsicherheit lässt sich daraus lernen, wie wichtig kollektive Achtsamkeit, Multiperspektivität, psychologische Sicherheit und Bereitschaft zu einem evolutionären Lernen sind.

## Fazit

In diesem Abschnitt sollen die wesentlichen Erkenntnisse des Artikels noch einmal gebündelt werden.

Nicht erst die Corona-Pandemie hat gezeigt, dass rationales Entscheiden unter VUCA-Bedingungen zunehmend an seine Grenzen stößt. Mit zunehmender Umweltdynamik wachsen Nichtwissen und fundamentale Unsicherheit, Systeme entziehen sich mit wachsender Komplexität einer linearen Steuerbarkeit. Anders als von der klassischen Theorie vorausgesetzt, können sich Entscheidungen in Organisationen in der Regel nicht an einem Ziel ausrichten, sondern müssen oft widersprüchlichen Erwartungen und Rationalitäten gerecht werden. In dieser Situation wachsen Unsicherheit, Stress und Angst auf der Seite der Entscheider*innen, da der Erwartungsdruck aus dem Umfeld trotz abnehmender Einflussmöglichkeiten oft aufrechterhalten bleibt.

Führungsentscheidungen müssen dazu beitragen, dass Organisationen im Spannungsfeld konfligierender Ziele ihre Existenz sichern, was nur gelingt, wenn sie ein gewisses Maß an Folgebereitschaft der Beschäftigten gewährleisten. Wo die Umsetzung angesichts begrenzter Machtmittel nicht erzwungen werden kann, müssen Entscheider*innen daher immer auch auf die Anschlussfähigkeit von Entscheidungen achten. Die Corona-Pandemie hat gezeigt, welche Herausforderungen sich hier stellen: Gerade in hochgradig unsicherheitsbehafteten Situationen ist eine professionelle Kommunikation von großer Bedeutung. Hier wurden die Empfehlungen zur Krisenkommunikation, das Modell der Salutogenese und das Konzept der Change Story als hilfreiche Instrumente genannt.

Ein weiterer für das Akzeptanzmanagement von Entscheidungen wichtiger Aspekt ist Vertrauen. Angesichts unklarer Zukunftsperspektiven und der Abhängigkeit von den Entscheidungen des Führungspersonals bedarf es einerseits eines hohen Maßes an Vertrauen in die fachliche Kompetenz und persönliche Integrität der Personen, die die Entscheidungen treffen. Im Kontext von Organisationen noch wichtiger erscheint allerdings das Systemvertrauen, d. h. der Glaube an die Funktionsfähigkeit von Institutionen und die regel- und verfahrensbasierte Entscheidungsfindung unabhängig von persönlichen Interessen und Urteilen. Im Artikel wurde ein Bezug zu den Forschungsbefunden zu prozeduraler Gerechtigkeit hergestellt.

Akzeptanz von Entscheidungen und Systemvertrauen hängen auch davon ab, auf welchem Wege Entscheidungen zustande kommen. Auch hier besteht ein Spannungsfeld: Auf der einen Seite braucht es eine angemessene Einbindung verschiedener Akteure in Entscheidungsprozesse, auf der anderen Seite muss es gerade in Krisensituationen möglich sein, Entscheidungen schnell und auch gegen Widerstände treffen zu können. Hier besteht in vielen Organisationen noch ein Defizit in Bezug auf eine systematische, differenzierte und transparente Zuordnung der Entscheidungsverfahren, die sich für unterschiedliche Entscheidungsanlässe am besten eignen.

Abschließend wurde anhand der HRO-Forschung geprüft, welche typischen Muster der Unsicherheitsbewältigung sich im deutschen Corona-Management beobachten ließen und welche allgemeinen Erkenntnisse sich daraus für Führen und Entscheiden unter Unsicherheit ableiten lassen. Hier wurde deutlich, dass ein immer ausgefeilteres System von Regeln nicht geeignet ist, um komplexe und dynamische Situationen zu bewältigen und dass es stattdessen einer Kultur der kollektiven Achtsamkeit bedarf, um Unsicherheit kompetent aufzufangen. Die Nutzung von Vielfalt als Ressource, die Stärkung der psychologischen Sicherheit und der Fokus auf soziales Lernen sind wichtige Entwicklungsfelder, die in vielen Organisationen noch intensiver bearbeitet werden müssen, um organisationale Resilienz für den Umgang mit künftigen Unsicherheiten auszubilden.

Die Corona-Pandemie zeigt: Organisationen werden sich ebenso wie die Gesamtgesellschaft auf zunehmende Unsicherheit einstellen müssen. Disruptionen in technologischer, wirtschaftlicher, sozialer oder gesundheitlicher Hinsicht sind keine „schwarzen Schwäne“ mehr. Organisationen können nicht im permanenten Krisenmodus existieren, sondern müssen daran arbeiten (und tun es vielfach bereits), Resilienzfähigkeiten für das Entscheiden unter Unsicherheit als neuer Normalität zu entwickeln. Um es mit den Worten der Verfassungsrichterin und Schriftstellerin Juli Zeh zu sagen: „Die Art, miteinander zu sprechen und zu entscheiden, darf nicht unter der suspendierenden Bedingung des Ausnahmezustands stehen. Andersherum gesagt, wir müssen unseren Normalitätsbegriff so normalisieren, dass er auch große Störfälle umfasst“ (Dorn et al. 2021).

Natürlich ist das Thema Entscheidungen unter Unsicherheit damit nicht abschließend behandelt. Viele relevante Aspekte (z. B. Angst, Stress und Resilienzfähigkeit auf der Seite der Entscheider*innen, Risikowahrnehmung, Einflüsse des organisationalen Kontextes, die Bedeutung von Mikropolitik auf Entscheidungsprozesse unter Unsicherheit, gruppendynamische Faktoren, organisationale Struktur- und Kulturmerkmale) konnten in diesem Beitrag nicht oder nur ansatzweise thematisiert werden. Wünschenswert wären vor allem eine intensivere interdisziplinäre Konzeptentwicklung und Forschungsaktivität zu Entscheiden unter Unsicherheit, um der steigenden Relevanz dieses facettenreichen Themas Genüge zu tun.

## Footnotes

1 Die im Beitrag verwendeten Beispiele beziehen sich auf die Corona-Politik und die öffentlichen Reaktionen darauf in Deutschland. In anderen Staaten (etwa Österreich und der Schweiz) ließen sich sicherlich ähnliche Dynamiken, aber auch Unterschiede beobachten. Dass diese nicht systematisch einbezogen wurden, hängt mit Platzbeschränkungen und mangelnder Detailkenntnis des Autors zusammen, vor allem mit dem Band von Gloger und Mascolo (2021), der anhand von Interviews einen Einblick in die Situation der politischen Entscheidungsträger*innen in Deutschland ermöglicht.

2 In der Literatur sind sehr viel differenziertere Konzepte von Unsicherheit beschrieben worden (vgl. etwa Dequech 2011), die für die Zwecke dieses Artikels allerdings nicht weiter ausgeführt werden müssen.

3 In diesem Zusammenhang ist die Frage diskutiert worden, ob die Anwendung des Paradoxiebegriffs auf die beschriebenen Spannungsfelder eine begriffliche Überdehnung darstellt. Hilfreich ist dabei z. B. die von Watzlawick eingeführte Unterscheidung zwischen logischen und pragmatischen Paradoxien, also solchen, die nicht auf einem logischen Widerspruch, sondern auf der Notwendigkeit beruht, unvereinbare Handlungsnotwendigkeiten zu vereinbaren. Begriffsfragen sollen hier nicht näher thematisiert werden, vgl. hierzu etwa Smith und Lewis (2011), S. 386 ff. oder Ortmann (2017).

4 Zu diesem Schluss kommt z. B. auch Aschauers empirische Studie zum gesellschaftlichen Unbehagen in der EU, vgl. Aschauer (2017), S. 591.
